# Adherence to changing from brand-name to generic atorvastatin in newly treated patients: a retrospective cohort study using health insurance claims

**DOI:** 10.1186/s40780-015-0013-8

**Published:** 2015-04-01

**Authors:** Yasunari Mano, Shota Fukushima, Hisayuki Kuroda, Hiroyuki Ohshima, Yoshinori Kato, Kaori Ohuchi, Kayoko Maezawa, Yasuyuki Momose, Shunya Ikeda, Mariko Asahi

**Affiliations:** Department of Pharmaceutical Sciences, International University of Health and Welfare, Otawara, Tochigi Japan

**Keywords:** Adherence, Proportion of days covered, Persistence, Generic substitution, Atorvastatin, Claim database, Cohort study

## Abstract

**Background:**

Effect of statin therapy has been reported to be associated with patient’s adherence. Atorvastatin was available in Japan as a brand-name product beginning in 2000. The first atorvastatin generics were introduced in Japan in November 2011. The objective of this study was to analyze whether changing from a brand-name atorvastatin to a generic product would affect patient adherence.

**Methods:**

We conducted a retrospective cohort study that included adult patients who received newly prescribed brand-name atorvastatin between June 1, 2011 and May 31, 2012, using a health insurance claims database in Japan. Patients were classified by the presence or absence of changing to a generic during the 6 months from December 1, 2011 to May 31, 2012 (the index period). The first prescription date for the generic or brand product during the index period was defined as the index date. Adherence to therapy was assessed by the proportion of days covered (PDC) and persistence of treatment by time to discontinuation.

**Results:**

There were 135 patients changing to generic atorvastatin and 147 continuing with the brand-name product. There was no significant difference in decrease of PDC from pre- to post-index date between the changed cohort and continued cohort (−8.6% vs −10.3%, respectively; *P* = 0.443). After adjusting for baseline covariates, including adherence in pre-index date, no statistically significant differences were observed in the adjusted odds of adherence between the cohorts (adjusted odds ratio = 0.83, 95% confidence interval (CI) = 0.46–1.53). There was also no significant difference in persistence between two cohorts in the 180-day after post-index date. After analysis of a Cox proportional hazard regression model controlling for baseline covariates, including adherence in pre-index date, no statistically significant differences were observed for the hazard of non-persistence between the cohorts (adjusted hazard ratio = 0.96, 95% CI = 0.60–1.53).

**Conclusions:**

Changing from a brand-name atorvastatin to generic product did not affect adherence for patients newly treated with atorvastatin.

## Background

The use of generic medicines has been increasing in recent years. Generic medicines are typically 20 to 90% cheaper than the brand-name equivalent [1]. Therefore, the substitution of brand-name medicines with generic equivalents can be a cost containment benefit for patients. Generic substitution has economic impacts in healthcare provision, in addition to the potential impact on the individual. For example, a study by the Generic Pharmaceutical Association showed that the use of generic medications in the American health system saved $239 billion in 2013 alone and generic products have saved the U.S. economy nearly $1.5 trillion over the past 10 years (2004–2013) [2].

Once a generic product has been approved based on bioequivalence, therapeutic equivalence is generally assumed. Therefore, there is no difference in safety and efficacy between the generic and original drugs [3]. However, some studies have reported that patients have concerns related to generic medicines. Patients felt that generic medicines did not work as effectively as the original medicine [4,5]. Shrank *et al.* found that a meaningful proportion of physicians expressed negative perceptions about generic medications, representing a potential barrier to generic use [6]. These concerns may result in decreased confidence in the treatment after generic substitution and may negatively affect patient’s adherence, which could result in less-effective treatment.

Atorvastatin is an inhibitor of 3-hydroxy-3-methylglutaryl coenzyme A reductase inhibitors (statin), which is effective in reducing low-density lipoprotein cholesterol and cardiovascular events [7,8]. It is approved for the treatment of hyperlipidemia in Japan. Atorvastatin was available in Japan as a brand-name product beginning in 2000. The first atorvastatin generics were introduced in Japan in November 2011. The effect of statin therapy has been reported to be associated with patient’s adherence [9-11]. However, no study has investigated whether changing from a brand-name atorvastatin to a generic product affect patient’s adherence.

The objective of this study was to analyze whether changing from a brand-name atorvastatin to a generic product would affect adherence in patients newly treated with atorvastatin using a health insurance claims database.

## Methods

### Study design and data sources

The study was designed as a retrospective cohort study using a health insurance claims database covering approximately 1,000,000 patients from the Japan Medical Data Center Co. Ltd (JMDC) in Tokyo, Japan [12]. This database provides information on individuals in employment-based health insurance programs, including demographic characteristics (eg age, gender), procedures, diagnoses of disease coded by International Classification of Disease, 10th Revision (ICD-10), and prescribed drugs (dose, quantity and number of days of supply) from ambulatory and inpatient care. Drugs are coded according to the Anatomical Classification of Pharmaceutical Products (ATC) of the European Pharmaceutical Market Research Association (EphMRA). The data of each individual can be tracked in chronological order if multiple medical institutions were used.

### Study population

We identified patients (aged ≥18 years) who received newly prescribed brand-name atorvastatin between June 1, 2011 and May 31, 2012. Newly prescribed patients were defined as those who did not have a statin prescription in the previous 6 months; prevalent users were excluded from this study.

The first atorvastatin generics were introduced in Japan in November 2011. Thus, we considered the presence or absence of changing to the generics during the 6 months from December 1, 2011 to May 31, 2012 (index period) and studied the influence of generics on adherence. The patients were considered part of the “changed cohort” if they changed from a brand-name atorvastatin to a generic product with bioequivalence during index period. The first prescription date of generic substitution of atorvastatin was defined as the index date. A change was defined as a prescription for a generic product within 30 days following the last day covered by the brand-name product [13]. During the index period, patients who did not change to a generic substitute, but continued with the use of the brand-name drug, were defined as the “continued cohort”; the index date for this cohort was defined as the first prescription date of the brand-name product during index period. All patients were continuously enrolled for a minimum of 6 months before and after the index date.

Patients were excluded in this study based on the following criteria.

1. The patient did not have a prescription for a brand-name product or did not change from brand-name to generic product during index period.
2. The patient had missing or invalid dates of supply or insufficient enrollment in the pre-index and post-index date.
3. There was a change in dosing schedule.
4. The patient did not meet the definition of changing (within 30 days).
5. The patient changed to other antihyperlipidemic agents or other antihyperlipidemic agents were added.

The study protocol was approved by the Institutional Review Board at International University of Health and Welfare Ethics Committee.

### Adherence

Adherence to therapy with atorvastatin was assessed by the proportion of days covered (PDC) and persistence of treatment by time to discontinuation.

The PDC was calculated as the number of days’ supply of medication divided by the number of days in the follow-up period for each patient [14]. PDC values were examined before the index date (baseline adherence) and after the index date. Baseline adherence was calculated from the first prescription within 180 days before the index date until the index date. The PDC after the index date was calculated during the 180-day follow-up period after index date. The supply for days falling beyond the 180-day study period was truncated and not used in the PDC calculation. Patients were considered adherent if PDC ≧ 80% [15]. Persistence was measured as the number of continuous days from index date to discontinuation in the 180-day [16]. Discontinuation was defined as a gap of greater than 30 days between medication supplies [17]. These methods were used in other studies of adherence [18-20].

### Other patient-related variables

The patient variables included age, gender, prescribing medical establishment (clinic, hospital), type of prescriber (general practitioner, cardiologist, or others), total number of drugs prescribed, other medications used for cardiovascular disease, and daily dosage of atorvastatin at the index date. Furthermore, comorbidities, the Charlson comorbidity index [21], which is a useful measure of comorbidities and health status were determined during 180 days prior to the index date using ICD-10 and ATC codes, and other medications for cardiovascular disease. The duration of use of atorvastatin prior to the index date was also examined.

### Statistical analyses

The demographic and clinical characteristics of patients in the changed and continued cohorts were compared using the χ^2^ test or Fisher’s exact test for categorical variables and Mann Whitney U test for continuous variables. Differences in PDC values, which included values before and after the index date, between the two cohorts were performed using Mann Whitney U test. Differences in PDC values from pre- to post-index date for the changed and continued cohorts were performed using paired t tests. Furthermore, the likelihood of being adherent (PDC ≧ 80%) was compared using an unadjusted logistic regression model, adjusted for baseline covariates, including baseline adherence (PDC ≧ 80%) and other variables that were significantly different between the two cohorts. Crude and adjusted odds ratios (OR) and 95% confidence intervals (CI) were estimated. Differences in persistence values were analyzed using Mann Whitney U test. Persistence rates were evaluated using Kaplan-Meier analysis, and the hazard of non-persistence in the cohorts was compared using a Cox proportional hazards regression model, adjusted by the same covariates as above. Crude and adjusted hazard ratios (HR) and 95% CI were estimated. Statistical significance was set at *P* < 0.05 in all analysis. SPSS 20.0 (SPSS Inc, Chicago, IL) was used for data analyses.

## Results

### Characteristics of the study population

**Figure 1:**
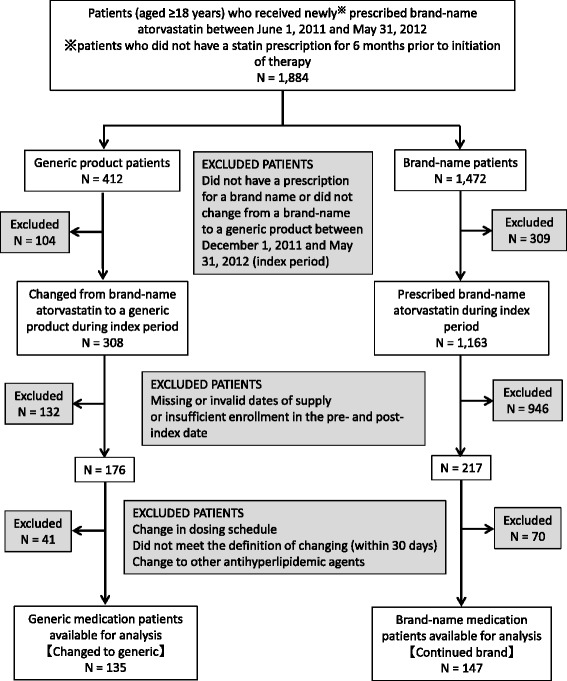
**Flow chart of the study cohort.**

In the 1,884 new users prescribed brand-name atorvastatin, there were 308 patients changing from a brand-name atorvastatin to a generic product during the index period, and 1,163 patients continuing the brand-name product during this period. After applying the exclusion criteria, 135 patients in the changed cohort and 147 patients in continued cohort were eligible for this study. Excluded patients of the continued cohort were so much than the generic cohort because many patients of the continued cohort did not have valid dates of supply in the pre- and post-index date (Figure 1).

Baseline characteristics of the study population are given in Table 1. The gender, age, and type of prescriber were similar between two cohorts, although there was significant difference in the prescribing medical establishment. There was no difference in the overall severity of comorbidities between the cohorts using the Charlson comorbidity index. However, the proportion of patients having hypertension did differ significantly between the cohorts (52.6% in the changed cohort vs 37.4% in the continued cohort; *P* < 0.05) and arrhythmia (0% vs 5.4%, respectively; *P* < 0.01). The total number of drugs prescribed was similar in the cohorts, and the use of other medications for cardiovascular disease was also similar, except in the proportion of patients treated with calcium channel blocker was significantly higher in the changed cohort than that in the continued cohort (22.2% vs 7.5%, respectively; *P* < 0.01). Almost all patients in this study received medications with a daily dosage of 5 mg or 10 mg. The median of duration of use of atorvastatin was significantly longer in the changed cohort than that in the continued cohort (126 days vs 98 day, respectively; *P* < 0.05).

**Table 1:** **Baseline characteristics of the study population**

**Characteristic** ^**a**^	**Changed to generic (n = 135)**	**Continued brand (n = 147)**	**P value**
**Gender**			
Male	78 (57.8)	84 (57.1)	0.914
Female	57 (42.2)	63 (42.9)	
**Age**	53 (27 ~ 73)	52 (25 ~ 72)	0.383
**Prescribing medical establishment**			
Clinic	83 (61.5)	111 (75.5)	<0.05
Hospital	52 (38.5)	36 (24.5)	
**Type of prescriber**			
General practitioner	51 (37.8)	59 (40.1)	
Cardiologist	5 (3.7)	3 (2.0)	0.841
Others	79 (58.5)	85 (5708)	
**Comorbidities**			
Depression	5 (3.7)	9 (6.1)	0.351
Cardiac disease	17 (12.6)	23 (15.6)	0.463
Cerebrovascular disease	18 (13.3)	15 (10.2)	0.415
Hypertension	71 (52.6)	55 (37.4)	<0.05
Diabetes mellitus	63 (46.7)	55 (37.4)	0.116
Cancer	21 (15.6)	22 (15.0)	0.891
Peripheral vascular disease	10 (7.4)	10 (6.8)	0.844
Arrhythmia	0	8 (5.4)	<0.01
Renal dysfunction	1 (0.7)	1 (0.7)	0.729
Dementia	0	1 (0.7)	0.521
**Charlson comorbidity index**	1.0 (0 ~ 12)	1.0 (0 ~ 13)	0.698
**Total number of drugs prescribed**	3 (1~25)	2 (1~26)	0.870
**Other mediation for cardiovascular disease**			
inotropic agent	1 (0.7)	2 (1.4)	0.532
Antiarrhythmic agent	1 (0.7)	1 (0.7)	0.729
Diuretics	6 (4.4)	6 (4.1)	0.880
β-Blocker	6 (4.4)	7 (4.8)	0.899
Calcium channel blocker	30 (22.2)	11 (7.5)	< 0.01
ACE inhibitor	1 (0.7)	1 (0.7)	0.729
Angiotensin II receptor antagonist	32 (23.7)	25 (17.0)	0.162
Direct Renin Inhibitor	0.0	1 (0.7)	0.521
Platelet aggregation inhibitor	9 (6.7)	16 (10.9)	0.213
**Daily dosage of atorvastatin**			
2.5 mg	1 (0.7)	0	
5 mg	66 (48.9)	61 (41.5)	
10 mg	68 (50.4)	82 (55.8)	0.224
15 mg	0	2 (1.4)	
20 mg	0	1 (0.7)	
30 mg	0	1 (0.7)	
**Duration of use of atorvastatin prior to index date** ^**b**^	126 (10~332)	98 (14~229)	< 0.05

*Abbreviation*: *ACE* angiotensin-converting enzyme.

^a^Values expressed as number (%) or median (range).

^b^Values of duration of use of atorvastatin prior to index date expressed as days.

### Proportion of patients achieving adherence

**Figure 2:**
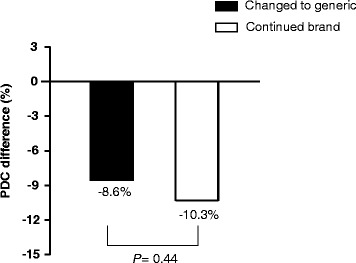
**Difference in proportion of days covered (PDC) from pre- to post-index date for the changed and continued cohorts.**

Table 2 shows the result of PDC of the two cohorts. In the pre-index date, the median of PDC value was significantly higher in the changed cohort than that in the continued cohort (97.7% vs 92.4%, respectively; *P* < 0.01). The proportion of patients achieving adherence (PDC ≧ 80%) was also higher in the changed cohort (85.9% vs 73.5%, respectively; *P* < 0.05). There was no difference in the median of PDC value in the post-index date between the two cohorts. Significant decreases in PDC change from pre- to post-index date were observed in the changed cohort (−8.6%) and continued cohort (−10.3%). However, the two cohorts had no statistically significant difference in PDC change (−8.6% vs −10.3%, respectively; *P* = 0.443) (Table 2 and Figure 2). The proportion of patients achieving adherence (PDC ≧ 80%) in post-index date was not significantly different (71.1% in the changed cohort and 63.3% in the continued cohort; *P* = 0.162; unadjusted odd ratio [OR] = 1.43; 95% confidence interval [CI] = 0.87–2.36). After adjustment for adherence in pre-index date (PDC ≧ 80%) and other covariates, such as patients characteristics and comorbidities at baseline, no statistically significant differences were observed for the adjusted odds of adherence between the changed and continued cohorts (adjusted OR = 0.84, 95% CI = 0.46-1.52).

**Table 2:** **Proportion of days covered in pre- and post-index date for the changed and continued cohorts**

**PDC**	**Changed to generic**	**Continued brand**	**P value**	**Odd ratio** ^**a**^ **(95 %CI)**	
				**Unadjusted**	**Adjusted** ^**b**^
**Pre-index date**					
PDC, %, median (range)	97.7 (34.1 ~ 100)	92.4 (22.9 ~ 100)	<0.01		
Patients achieving adherence (PDC≧80%) (number, (%))	116 (85.9)	108 (73.5)	< 0.05		
**Post-index date**					
PDC, %, median (range)	92.2 (7.8~100)	89.4 (15.6~100)	0.058		
Patients achieving adherence (PDC≧80%) (number, (%))	96 (71.1)	93 (63.3)	0.162	1.43 (0.87-2.36)	0.84 (0.46-1.52)
**PDC difference from pre- to post-index date (%)**	- 8.6^**^	- 10.3^**^	0.443		

Median of proportion of days covered (PDC) and proportion of patients achieving adherence (PDC ≧ 80%) in the pre- and post-index date, and odds ratio of achieving adherence (PDC ≧ 80%) for the changed and continued cohorts.

^a^Logistic regression model analysis.

^b^Adjusted baseline covariates included adherence in pre-index date (baseline adherence) (PDC≧80 %), duration of use of atorvastatin prior to index date, prescribing medical establishment, and comorbidities, such as hypertension, arrhythmia, and calcium channel blocker.

**:Significant difference in PDC change from pre- to post-index date for the changed and continued cohorts (*P* < 0.01).

### Persistence of treatment

**Figure 3:**
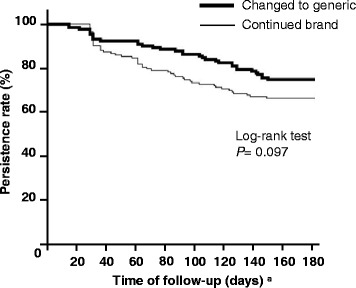
**Kaplan-Meier analysis of persistence with therapy for the changed and continued cohorts.** a: Time of follow up started from index date.

Table 3 shows the persistence of treatment in the two cohorts. There was no significant difference in persistence duration (time to discontinuation) between the two cohorts in the 180-day post-index date. Persistence rates in the two cohorts gradually fell to 75.6% and 67.3%, respectively, at 180 days and no significant difference was observed (*P* = 0.097, Log-rank test; unadjusted hazard ratio [HR] = 0.69; 95% CI = 0.44–1.08) (Figure 3 and Table 3). After analysis of the Cox proportional hazard regression model, which controlled for adherence in pre-index date (PDC ≧ 80%), baseline characteristics, and comorbidities, no statistically significant differences were observed for the hazard of non-persistence between the changed and continued cohorts (adjusted HR = 0.97, 95% CI = 0.61–1.55).

**Table 3:** **Persistence duration and associated hazard ratio for the changed and continued cohorts**

**Persistence duration** ^**a**^ **, days, median (range)**			**Hazard ratio** ^**b**^ **(95 %CI)**	
**Changed to generic**	**Continued brand**	**P value**	**Unadjusted**	**Adjusted** ^**c**^
180 (14~180)	180 (28~180)	0.076	0.69 (0.44-1.08)	0.97 (0.61-1.55)

^a^Persistence was measured as the number of continuous days from index date to discontinuation in the 180-day.

^b^Cox proportional hazard model.

^c^Adjusted baseline covariates included adherence in pre-index date (baseline adherence) (PDC≧80 %), duration of use of atorvastatin prior to index date, prescribing medical establishment, comorbidities such as hypertension, arrhythmia, and calcium channel blocker.

### Sensitivity analysis

A sensitivity analysis was conducted to assess differences in adherence for patients who were “not adherent” at baseline (PDC was < 80% in the pre-index date) in each cohort. There was no significant difference in the proportion of patients achieving adherence between the changed and continued cohorts (adjusted OR = 1.00, 95% CI = 0.23–4.27). Persistence with therapy was not significantly different (adjusted HR = 0.92, 95% CI = 0.38–2.19).

## Discussion

This study suggested that medication adherence for patients newly treated with atorvastatin was not negatively influenced by changing from a brand-name atorvastatin to a generic product. In fact, there was no significant difference in the proportion of patients achieving adherence between those changing to a generic drug and those continuing with a brand-name drug. Furthermore, there was no difference in the persistence with therapy across the cohorts.

A number of generic statins were approved, and the rate of changing to generic statins increased. Chapman *et al.* [13] showed that therapeutic substitution patients, who changed from a brand-name statin to a generic version of a different statin, were 33% less likely to be adherent compared with generic substitution patients, who changed from a branded to a generic version of the same statin at 6-month post-change. However, there are few reports about adherence with changes from a brand-name statin to a generic version of same statin compared with continuing with the brand-name statin. Gagne *et al.* [22] showed that patients treated with generic statins were more likely to adhere and had a lower rate of composite clinical outcome compared with those treated with brand-name version of same statin. This discrepancy in adherence between our finding and this study might be due to situations regarding the health insurance and socioeconomy in the United States and Japan. The study cohort comprised Medicare beneficiaries aged 65 years or older. Other potential reason for this discrepancy might be study design. We designed taking into account changing from a brand-name to generic product, however the study compared patients initiating generic statins with those initiating brand-name products. For the treatment of hypertension, some studies [23,24] have compared changing to a generic substitute with continuing brand-name drugs; these studies showed that the generic substitution of an antihypertensive drug did not lead to lower adherence. Our findings were consistent with these studies. Studies about generic substitution for new users who are therapeutic initiators, but not prevalent users, are limited [23]. Prevalent users who have been using statins for long time tend to be healthier and more adherent to therapy and screening than new users [15,25]. This phenomenon may result in biased estimates of the effect of adherence. Thus, we examined new users. This study of adherence in new users when changing from a brand-name statin to a generic product of same statin adds to the literature.

Adherence to statin treatment was associated with health outcomes, such as reduction in major vascular events, lower cardiovascular morbidity and mortality rates [9-11]. Our finding may imply that incidence of health outcomes is not increased by the change from a brand-name atorvastatin to a generic product. Thus, changing to a generic product may have health benefits in addition to the known cost benefits. Further research is required to confirm the effect on health outcomes following the change to a generic product.

The PDC values were expressed as median and range in our study. The mean PDC values in two cohorts ranged from was 77.6% to 83.3% in the post-index date (data not shown). These results differed from those of Benner *et al.* [15] who found the mean PDC was 56% after the first 6 months of statin treatment. This might be due to a difference in the age of population. The patients in our study were middle-aged (mean age = 52), whereas those in Benner *et al.* were elderly (mean age = 74 years). However, the persistence rate 6 months after the index date fell to an average of 67.3% and 75.6%, which is comparable to the study of Perreault *et al.* [26]. They found a 65% persistence rate in a group of middle-age patients, also newly treated with statins, after the first 6 months of treatment. Further research is required to analyze whether various kinds of generic atorvastatin affect patient adherence.

The limitations of this study include the potential for the several forms of bias. Due to the retrospective and non-randomized nature of the design, selection bias may have resulted in more adherent patients in the changing cohort at baseline. In the pre-index date, the proportion of patients achieving adherence (PDC ≧ 80%) among the changed cohort was higher than that of the continued cohort (85.9% vs 73.5%). These rates may be influenced by physicians; physicians may not change patients from a brand-name product to a generic product if the patient has poor adherence, or it is assumed that a change might negatively affect the treatment. However, the result of the sensitivity analysis for non-adherent patients at baseline in the two cohorts found no significant difference in adherence. There were some differences in patient characteristics between the cohorts, including the duration of use of atorvastatin prior to the index date, prescribing medical establishment, and comorbidities, such as hypertension, arrhythmia, and other medications, such as calcium channel blockers. Although there may be covariates that we did not include, we adjusted for the known covariates included baseline adherence in pre-index date to reduce the influence of any bias.

Another limitation in our study may have introduced information bias. Analysis of adherence was based on pharmacy dispensing records, and the actual medication taken by the patients is unknown. This unknown may affect the cohorts because it is probably non-differentially distributed between the two groups. However, most studies using the validity of prescription rates have found that measures of prescription rates are significantly associated with clinical measures of adherence, serum drug levels, or physiologic drug effects [27].

There were other potential confounding factors that could not be considered in this insurance claim database analysis. Information on adverse experiences and laboratory tests was not available. Furthermore, medical counseling, the patient’s beliefs about treatment, and the effects on clinical end-points, such as measures of cholesterol, were not included in the database. Although studies using information from such databases can access information on many patients gathered in real-world conditions, it is not possible to assess the influence of these other factors. However, the strengths of this study include the available factors related to adherence, such as prescribed daily doses and diagnoses, which minimized any confounding bias. Furthermore, the claim database included few elderly patients. Therefore, generalization of these results to elderly patients is limited.

Although generic products are not still fully covered in Japan, patients are encouraged to accept changing from a brand-name drug to a generic product from the perspective of adherence and cost.

## Conclusions

In conclusion, the results of this study suggest that changing from a brand-name atorvastatin to a generic product does not lead to a decrease in adherence for patients newly treated with atorvastatin. Therefore, changing to generics for patients seems to be reasonable from the perspective of adherence to statin therapy and cost benefits. Further investigation is required to confirm these results with other types of medications for chronic conditions.
